# Short-Term Follow-up of Surgical Management Verruca Vulgaris with Modified Estlander Flap: A Case Report and Recent Literature Review

**DOI:** 10.4317/jced.61814

**Published:** 2024-08-01

**Authors:** Mohammad Gazali, Muhammad Ruslin, Carolina Stevanie, Andi-Sitti-Hajrah Yusuf, Aisha A. H. Al-Jamaei, Paolo Boffano, Tymour Forouzanfar, Kei Tomihara

**Affiliations:** 1 Department of Oral and Maxillofacial Surgery, Faculty of Dentistry, Hasanuddin University, Makassar, Indonesia; 2 Department of Oral Surgery and Oral Medicine, Collage of Dentistry, Al-Razi University, Sana’a, Yemen; 3 Department of Dentistry, AOU Maggiore Della Carità, Novara Italy; 4 Department of Health Sciences, University of Eastern Piedmont, Novara, Italy; 5 Department of Oral and Maxillofacial Surgery/Oral Pathology, Amsterdam UMC, the Netherlands; 6 Department of Oral and Maxillofacial Surgery, Leiden University Medical Center, Leiden, the Netherlands; 7 Divisions of Oral and Maxillofacial Surgery, Niigata University Graduate School of Medical and Dental Science, Niigata, Japan

## Abstract

**Background:**

Verruca vulgaris (VV), widely known as warts, is a common benign skin lesion, which is caused by human papilloma virus. In some cases, VV can be developed within the oral cavity. Surgical excision is considered as the most preferred treatment modality for both cutaneous and oral VV which could be challenge to deal with.

**Case Report:**

Herein, a short-term case of a 64-year-old male patient with a large oral VV, involving the lower and upper lips, and commissure was reported. The patient underwent a wide surgical excision, resulting in a significant lip defect. The modified Estlander flap technique was applied to treat the defect and restore lip function. At one year post operation, no signs of recurrence were recorded, and the Estlander flap technique displayed satisfactory outcomes.

**Conclusions:**

Surgical management of oral VV involving lips may leaves large defect, which requires consideration in defect reconstruction. This case report shows that reconstruction of the defect with a modified estlander flap resulted in a good outcome, with satisfactory functionality for the patient.

** Key words:**Estlander flap, verruca vulgaris, surgical excision.

## Introduction

*Verruca vulgaris* (VV) is a benign epithelial tumor, which is caused by human papilloma virus (HPV) infection, with HPV-2, HPV-4 or HPV-40 being the most commonly implicated causative agents (1-3). VV is a quite common disorder of the skin and relatively uncommon intraorally (4,5). According to the contagious nature of this disorder, oral lesions can also occur via autoinoculation (6,7). Although oral VV can affect any surface of the oral cavity, it is commonly presented as localized lesions on the palate, tongue and lips (8). Clinically, VV is characterized by an exophytic lesion with hyperkeratotic surface, ranging from 1 mm to >1 cm in size, commonly asymptomatic, slow-growing and can lead to cosmetic disFigurements (5,9).

Several treatment options are available for treating VV lesions, including surgical excision, cryotherapy, electrocauterization, laser therapy and the use of topical medications (10,11). The above treatment options aim to alleviate VV signs and symptoms. However, VV lesions identified as “recalcitrant”, that sometimes resistant to an initial treatment method and requiring alternative surgical therapy (12,13). Of note, the number of studies on the surgical management of extensive excision of oral VV following defect reconstruction in older patients is limited. Therefore, the current study reported a case of an elderly patient with oral VV, extending from the lips to the oral mucosa article, who underwent surgical excision and lip reconstruction using a modified Estlander flap.

## Case Report

Herein, a case of a 64-year-old male patient, who presented with a verrucous growth on his right lower lip skin, extended to the mucosa, which had been growing progressively over the past three years, was reported. The growth had a firm consistency and the patient remained asymptomatic during this period. Notably, no palpable lymph nodes were detected in the neck region. The patient was an active smoker, while he had no history of betel nut chewing and family history of similar diseases. Additionally, the patient suffered from uncontrolled hypertension.

**Figure 1:**
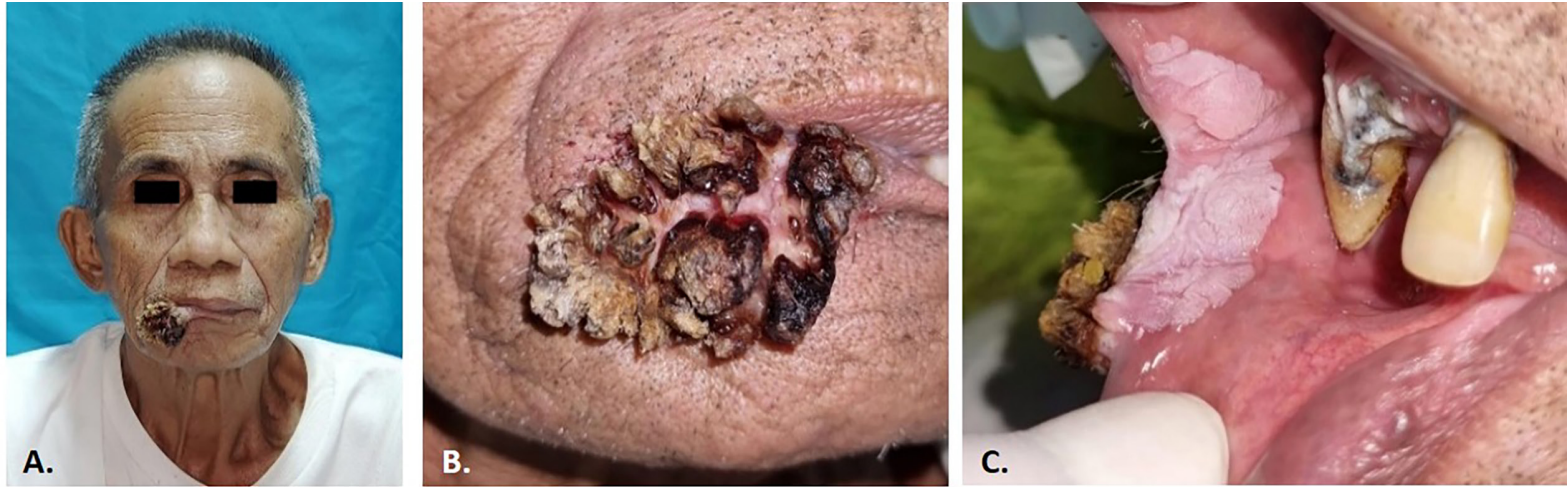
Pre-operative appearance A.) A proliferative verruca lesion appeared of the right lower lip that extended to the right upper lip. C.) Plaque-like lesions on the mucosa of the right corner lip.

Clinical examination revealed a verrucous proliferative growth on the right lower lip, extending to the right upper lip, involving the right commissural mucosa. The extra-oral lesion appeared exophytic and firmly adherent to the underlying tissues, with a size of 4x3x2 cm. Its surface was irregular and firm with blackish color. The base of the lesion displayed yellowish-white coloration and spongy texture, while it was not accompanied by pain or unusual sensations as shown in (Fig. 1).

Within the oral cavity, a well-demarcated, painless, white plaque-like lesion, 3x2x0.5 cm in size was observed on the mucosa of the right corner lip, which could not be detached and did not bleed easily. No similar lesions were found on the other parts of the body. Based on clinical presentation and examination, a provisional diagnosis of VV was established.

**Figure 2:**
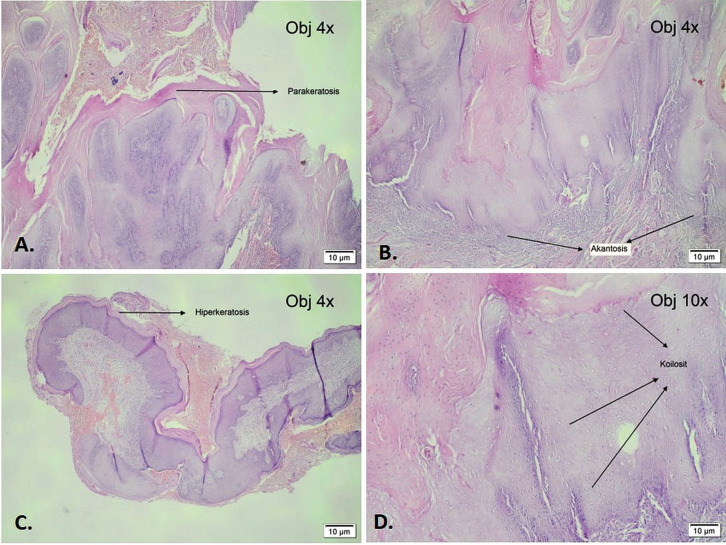
Histopathological examination of post-operative tissues showing Parakeratosis, hyperkeratosis and acanthosis tissues (A,B,C). Koilocytes were seen at 10 times microscopic magnification (D).

For further histopathological examination, a biopsy of the lesion was performed, which showed a fibrovascular peduncle tissue covered by hyperkeratotic, hypergranulated, papillomatous and acanthotic squamous epithelium (Fig. 2A,B). Therefore, the patient underwent planned definitive surgical procedure, including a wide excision and subsequent lip reconstruction using a modified rotational flap technique.

**Figure 3:**
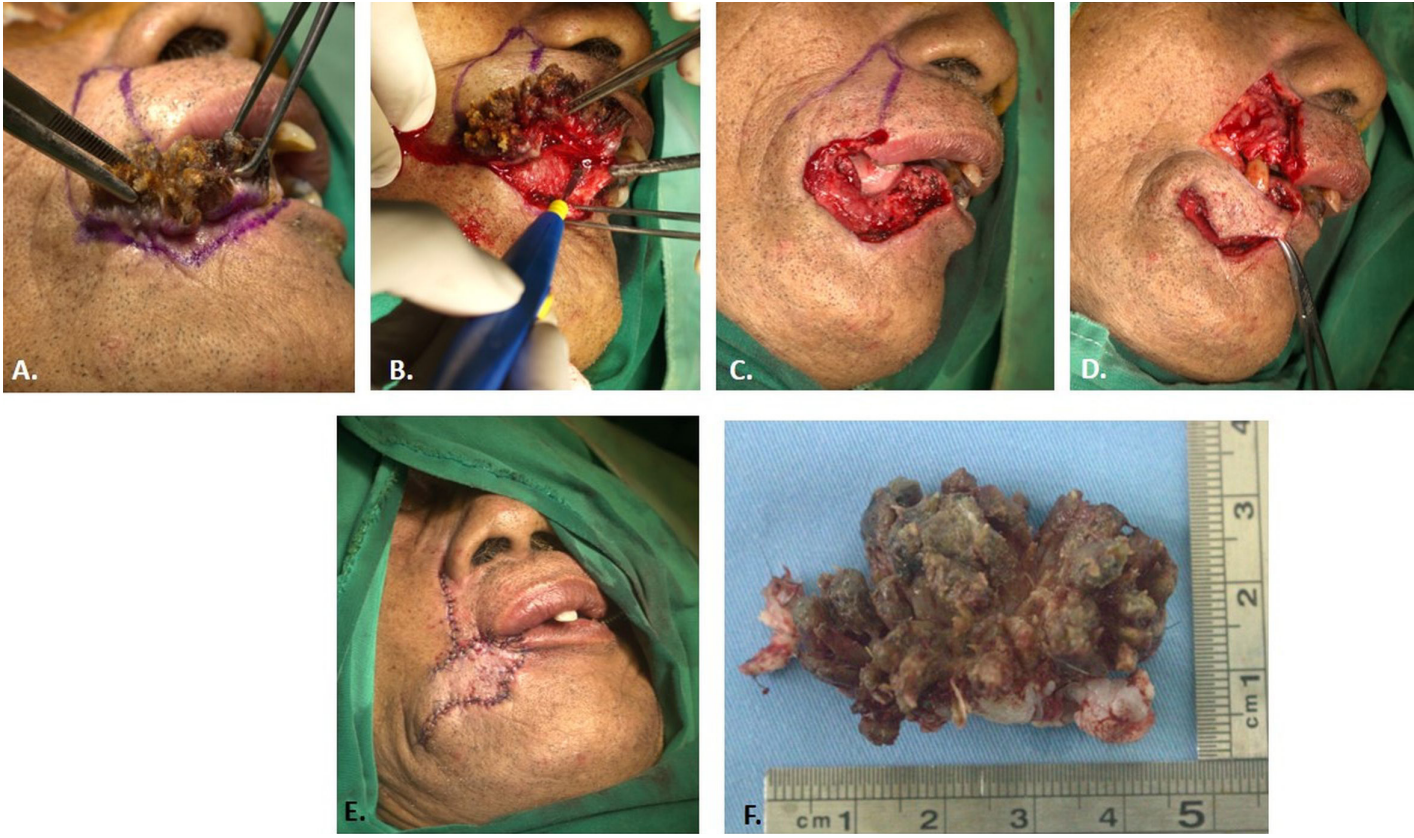
Intra-operative findings A.) Marking flap is created along the side of the tumor extending to the nasolabial line and lateral margin of the right nostril. B.) Full thickness tumor excision ± 2mm from the tumor border according to the marking, bleeding control was performed using a cautery. C.) Post excision surgery area. D.) Lip reconstruction with modified Estlander flap. E.) Post surgery. F.) Tumor mass.

The surgical marking covered both the areas of the lesion and extended to the nasolabial line. The full-thickness incision was extended to the inner mucosal layer and then to the nasolabial line area, following the marking delineating the healthy skin area, that was used as a flap for lip reconstruction. During surgery, granulomatous tissues were observed. Subsequently, the flap was placed on the right alignment to close the defect and was then dissected to avoid excess tension, as needed. Finally, a simple interrupted suture was performed to complete lip reconstruction as shown on Fig. 3.

The tumor mass was then subjected to histopathological examination. The assessment of the tissue specimens revealed an epidermis with hyperplasia. Additionally, further evident features, such as hyperkeratosis, acanthosis with papillomatous structure and an elongated rete ridge directed toward the center, were observed. Furthermore, koilocytes were also distinctly identified. In the dermis layer, a dense scattering of inflammatory cells, including lymphocytes and histiocytes, was observed. The above histopathological findings unequivocally verified the diagnosis of VV (Fig. 2C,D).

**Figure 4:**
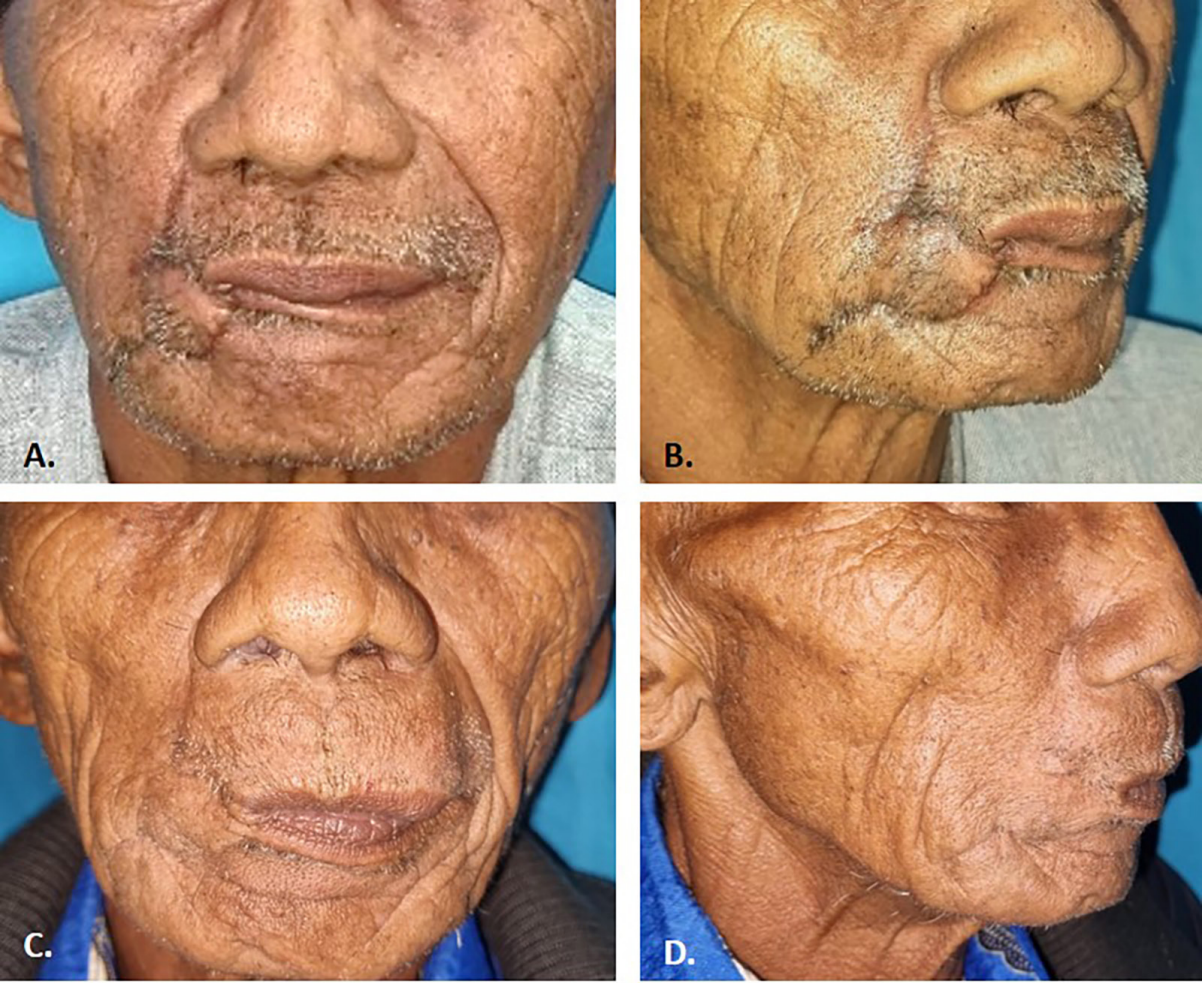
Follow-up post operation. A) 21-day Frontal view, right lip appears asymmetrical. B) Oblique view (45º), there is a scar on the postoperative area. C) Follow up on 1 year post operation. D) Oblique view shows good healing with minimal scar tissue.

On the three-week follow-up, limited mouth opening and asymmetry in the lips were recorded. The patient did not complain of pain and could clearly speak. The surgical wound was normal, with no indications of infection. At one year follow-up, the patient reported no pain and other functional limits. The mouth opening reached 40 mm, accompanied by normal lip movement. The patient was satisfied with the result and no sign of recurrence was observed (Fig. 4).

## Discussion

VV, widely known as warts, is a skin disorder caused by HPV infection. It affects ~7-10% of the general population (7,14). These lesions are commonly found on the skin of the fingers (most common), hands, knees and elbows (7,9). Within the oral cavity, VV is presented as a pink to white lesion, which is sessile, >1 cm in size with exophytic fronds (4,12). These lesions can result from the invasion of HPV into epithelial cells, thus promoting cell proliferation and growth/plaque formation (7).

Minor abrasion of the macerated area can serve as the entry point for HPV into the basal keratinocyte layer of the epithelium (7). Warts could be considered as contagious disorders, that could be transmitted through direct or indirect contact. Transmission can also be promoted by autoinoculation, which frequently occurs due to scratching, shaving or skin trauma, in general (7,9,15).

Oral VV commonly grows slowly and can remain unchanged in size for years. Importantly, although some lesions can shrink or heal on their own, depending on the host’s immune response, others can potentially enlarge and become reluctant to topical treatments (7-9).

The examination of the cervical lymph nodes is mandatory to rule out metastasis or malignancy (16). No palpable and painless findings upon palpation suggest that there is no metastasis to the cervical lymph nodes and the tumor is likely to be benign (10,16).

Histologically, oral VV exhibits an acanthotic epidermis with papillomatosis, hyperkeratosis and parakeratosis (17). Elongated ridges commonly curve toward the center of the wart. Skin capillaries are prominent and can be thrombosed (3). Lesions infected with HPV are characterized by large keratinocytes with eccentric pyknotic nuclei surrounded by a perinuclear halo (koilocytes) (2,3). These cells may have small eosinophilic granules and diffuse clumps of basophilic keratohyalin granules (2,3,8).

In several cases, oral VV does not require treatment and ~52% of cases resolve within 11-18 months without intervention (4,11). Persistent or larger lesions may require surgical removal either by routine excision or laser ablation (5,14).

Surgical excision has been adopted as a first line therapy for VV, with a success rate of ~65-85% (4,7). The management of these lesions is critical for minimizing virus propagation and preventing the formation of malignant lesions within the oral cavity (4). For large lesions, wide excision and careful reconstruction of the lips are suggested to minimize the risk of recurrence and achieve optimal functional and aesthetic outcomes (8,18,19).

The lips are the central point of the face, since they are significantly involved in expression and communication (20). The objectives of lip reconstruction are the restoration of oral competence, followed by the proper anatomic location, maintenance of muscular continuity, sensation and circumference of the oral aperture (21). Herein, the lesions involved both the mucosa and skin. To avoid microstomia, it is critical to maintain the border between the vermilion and normal skin (Klein’s line) and correctly position the commissures (limited mouth opening) (18,20).

Notably, the Estlander flap is commonly recommended when the labial defects encompass the commissure (25). In this particular case, a modified Estlander flap method, including the preparation of a triangle on the upper lip, was applied to reconstruct the lower lip and commissure. This was a full-thickness flap, which could cause distortion and shortening of the lower lip commissure, ultimately resulting in limited mouth opening (16,22).

It has been reported that the application of Estlander flap can be modified based on the location of the defect to better meet the reconstruction needs (20). For example, Marco *et al*. (23) utilized Estlander flap for lip reconstruction in a 71-year-old patient with basal cell carcinoma on the right cheek, who underwent surgical incision. At two years after surgery, satisfactory function and aesthetic outcomes were recorded (23). Consistently, Chihiro *et al*. (24) performed a similar procedure in a 70-year-old patient with buccal mucosal squamous cell carcinoma, accomplishing successful tumor resection, with lip and commissure reconstruction. At 24 months follow-up, the patient was able to speak properly and the lip sensation recovery was optimal.

This modified Estlander flap technique could provide a single-stage solution for reconstructing defects involving the lower lip and commissure (22). In terms of patient age, performing the incision within the structural boundaries could prevent scar formation and effectively restore the cosmetically sensitive, neurotized and functional tissue (18,20,25).

Despite its advantages, this technique poses risks and complications, such as infections, inflammation and limited mouth opening. Enteral and parenteral nutrition may be needed at some stages to maintain patent nutrition (18,20).

In a previous study, to ensure the success of the reconstruction, the patient was advised to maintain proper dental health and avoid smoking (10) Wound dressing and bandages are used to cover the incision sites, to limit sun exposure and prevent contamination. The bandage is typically changed once a day in such cases. Once incisions have healed, the sutures are removed and antibiotic ointment can be applied. However, when scars are developed or become pigmented after the initial healing phase, vitamin C-containing ointments could be applied to improve the appearance of the scars (25).

-Recent literature review

There are several management strategies for oral VV, depending on the size, location and age of the patient ( Table 1). In elderly patients, surgical excision is considered as the most appropriate treatment strategy, since it is safe and effective in removing the lesions. Tebcherany *et al*. (12) successfully managed a 71-year-old patient with VV at the junction of the soft and hard palate via performing an excisional biopsy under local anesthesia. At six months after surgery, no recurrence was recorded, while the area appeared normal.

Khoo *et al*. (23) also applied surgical excision to effectively remove a verruca lesion in the torus palatinus of a 48-year-old patient. The above patient also underwent thorectomy and no recurrence was recorded after six-month follow-up.

Additionally, Bouguezzi *et al*. (24) reported the case of a verrucous tissue growth on the lip mucosa of a 23-year-old patient. The lesion was excised, and no recurrence was reported at one year after surgery (27). Consistently, Mattoo *et al*. (8) reported the case of a solitary proliferative verrucous growth on the buccal mucosa, which was also treated with surgical excision. At one year after the operation, no recurrence was noted.

Emerging evidence has also suggested that VV can recur, particularly in association with several factors, including age, virus re-infection, subclinical HPV DNA immune status and smoking (4,28). Therefore, the high risk of recurrence in smokers has been associated with the chronic effects of smoking, which can eventually result in inflammation and diminished immune responses (25). To emphasize, previous studies also indicated that the association between human macrophages and extracellular matrix protein could change due to smoking, thus attenuating the ability of macrophages to phagocyte apoptotic neutrophils (28,29).

In cases of recurrent VV lesions, topical agents could be the treatment of choice. Ruiz-Huertas *et al*. (17) demonstrated a successful management of post-excision VV recurrence in a 74-year-old female patient, using a topical agent, namely Imiquimod (concentration, 15%). The authors reported that the lesions disappeared after the application of imiquimod for 16 weeks (17). However, when used on the skin, imiquimod can cause burning sensations, discomfort, erythema, and depigmentation, similar to vitiligo (11).

## Conclusions

The surgical management of oral VV in the elderly is challenging, since the aging process can alter the structure of the skin and that of facial muscles, thus making surgical excision a potential risk to the patient’s healing process.

The current study reported a short-term case of VV on the lip of an elderly patient. The lesion was completely excised and lip reconstruction was successfully applied using the modified Estlander flap method. In addition, effective surgical wound care and regulation of the patient’s smoking habits contribute to satisfactory outcomes in the management of oral VV. Considering the risk of low skin elasticity and vascularity in elderly patients, the outcomes in this case showed good healing and functional recovery rates. Eventhough the limitation of mouth opening was present, the patient was satisfied with the outcomes without complains of his mouth functionality.

## Figures and Tables

**Table 1:** Recent literature management of oral verruca vulgaris.

No.	Author	Year	Gender and age	Clinical findings	Intervention	Outcome
1	Tebcherany, et al. (12).	2022	Male (71)	An irregular outlined lesion at the junction of the soft and hard palate on the right side. The lesion raised rough pink to white plaque with a verrucous surface and ill-defined margin. It measured 6 x 6mm, hard on palpation and sessile fixed-based.	Excisional biopsy under local anesthesia.	Clinical follow-up at six months postoperatively showed complete tissue healing with no signs of recurrence.
2	Ruiz-Huertas, et al. (17).	2022	Female (74)	Recurrent of oral florid papillomatosis on lower lip – post excision	Application of topical Imiquimod 5%, three times a week.	Complete disappearance of the lesion after 16 weeks usage of imiquimod 5%.
3	Khoo et al., (26).	2021	Male (49)	A whitish lesion confined to the middle of a torus palatinus. Asymptomatic and not tender on palpation.	Excisional biopsy and torectomy under local anesthesia.	14 days after surgery, the site had healed uneventfully. At 6 months follow-up revealed no recurrence.
4	Bouguezzi, et al. (27).	2020	Male (23)	A solitary proliferative verrucous growth on the labial mucosa. The lesion was exophytic and sessile with irregular margin. surface of the lesion was irregular at the periphery with finger-like projection. The color was white and soft in consistency.	Excisional biopsy under local anesthesia.	Two weeks follow-up, the patient was completely asymptomatic, and no reccurence had been seen at the 1-year follow-up.
5	Mattoo, et al. (8).	2017	Male (48)	A solitary proliferative verrucous growth on the left buccal mucosa. Exophytic lesion and sessile, approximately 2 x 3 cm in size, irregular margin, the surface of the lesion was irregular at the periphery with finger-like projection at the center.	Excisional biopsy under local anesthesia.	The clinical follow-up showed complete tissue healing, with no recurrence at the 1-year follow-up.

## Data Availability

The original contributions presented in the study are included in the article/supplementary material. Further inquiries can be directed to the corresponding authors.
